# Optimization of cerebral organoids: a more qualified model for Alzheimer’s disease research

**DOI:** 10.1186/s40035-021-00252-3

**Published:** 2021-08-09

**Authors:** Feng-Chen Bi, Xin-He Yang, Xiao-Yu Cheng, Wen-Bin Deng, Xiao-Li Guo, Hui Yang, Yin Wang, Juan Li, Yao Yao

**Affiliations:** 1 School of Basic Medical Sciences, Ningxia Medical University, Yinchuan, 750004 China; 2 Key Laboratory of Traditional Chinese Medicine Modernization, Ministry of Education, Ningxia Medical University, Yinchuan, 750004 China; 3 School of Pharmacy, Ningxia Medical University, Yinchuan, 750004 China; 4 Department of Neurology and Suzhou Clinical Research Center of Neurological Disease, The Second Affiliated Hospital, Soochow University, Suzhou, 215004 China; 5 School of Pharmaceutical Sciences (Shenzhen), Sun Yat-sen University, Guangzhou, 510275 China; 6 Research Center of Medical Science and Technology, Ningxia Medical University, Yinchuan, 750004 China

**Keywords:** Cerebral organoids, Alzheimer’s disease, Pluripotent stem cells

## Abstract

Alzheimer’s disease (AD) is a neurodegenerative disease that currently cannot be cured by any drug or intervention, due to its complicated pathogenesis. Current animal and cellular models of AD are unable to meet research needs for AD. However, recent three-dimensional (3D) cerebral organoid models derived from human stem cells have provided a new tool to study molecular mechanisms and pharmaceutical developments of AD. In this review, we discuss the advantages and key limitations of the AD cerebral organoid system in comparison to the commonly used AD models, and propose possible solutions, in order to improve their application in AD research. Ethical concerns associated with human cerebral organoids are also discussed. We also summarize future directions of studies that will improve the cerebral organoid system to better model the pathological events observed in AD brains.

## Background

Alzheimer’s disease (AD) is a neurodegenerative disease characterized by progressive memory deterioration, cognitive disorders, and loss of independence. AD is a common disease in the elderly with a high fatality rate, secondary only to infections and malignancies. According to the 2018 global data from the International Alzheimer’s Disease Association, AD has affected 50 million people worldwide, and this number will rise to 82 million by 2030 and to 152 million by 2050. The report also identifies a considerable need for financial resources, goods and services, and long-term care for AD patients. The report also estimates the socio-economic burden of AD to be approximately $1 trillion in 2018 and be increased to $2 trillion by 2030. To date, there is no cure for AD, so both AD prevention and treatment remain urgently needed in the global medical community [1].

As the existing animal models of AD cannot fully recapitulate the pathophysiology in human patients, it remains difficult to effectively evaluate the efficacy and mechanisms of AD drugs. This is the main reason for the failure of new AD drugs in phase III clinical trials, which, however, demonstrated good therapeutic effects in early animal-model studies.

In recent years, 3D cerebral organoid cultures, established using the stem cell technology, have shown significant advantages in modeling nervous system diseases over the animal and cellular models. This increase in modeling precision has set new standards for the study of nervous system diseases, including AD. Although there have been some reviews on the cerebral organoids technology, there is still a lack of summary focusing on the AD mini-brain. In the following, we will discuss the advantages and disadvantages of AD cerebral organoids and introduce new technologies for cerebral organoid modification. Furthermore, potential schemes or directions for optimization of the current AD cerebral organoid models are proposed, in order to provide a new approach for AD research.

### Pathology and pathogenesis of AD

**Fig. 1:**
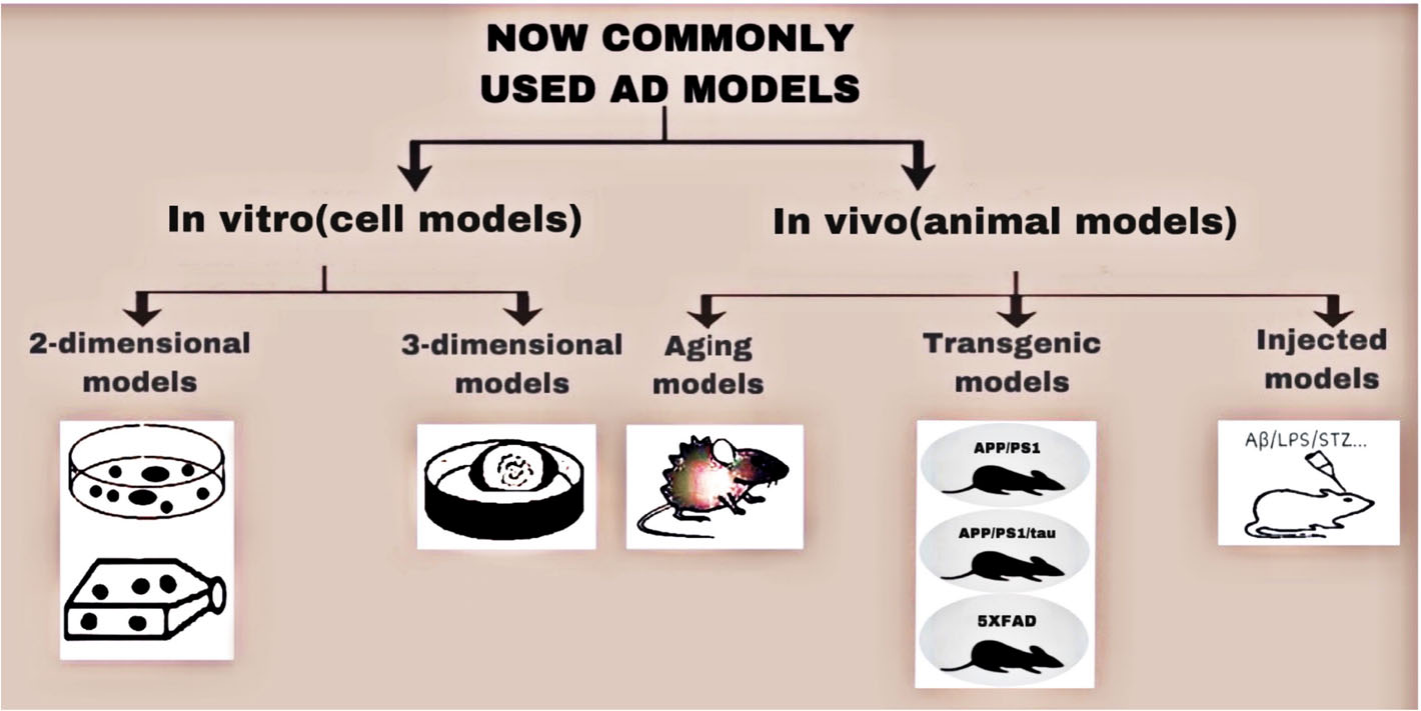
Commonly used animal- and cell-based AD models. Animal models, mostly rodent-based, can be divided into three categories: aging models, transgenic models, and models established by substance injections. Induced neurons or co-cultures of neurons and glial cells are commonly used as AD cell models but suffer from restrictions, such as the non-physiological top-to-bottom polar distribution of neuron-glial cells, and a lack of synaptic connections between cells. The 3D culture conditions may overcome these limitations

Protein misfolding is a hallmark of AD, involving the accumulations of amyloid-β (Aβ) peptide and hyperphosphorylated tau protein in discrete areas of the brain. These lesions are proposed to be a triggering step for a cascade of molecular events resulting in neurofibrillary tangles (NFTs), synaptic dysfunction, neuronal degeneration, and neuroinflammation, leading to both cognitive and memory impairments. Although NFTs are also observed in other neurodegenerative diseases such as cortical basal degeneration and frontotemporal dementia, amyloidosis is most commonly found in AD. AD can be categorized into familial AD (FAD) and sporadic AD (SAD). Most FAD cases are caused by mutations in genes encoding amyloid precursor protein (*APP*) and presenilin 1 (*PSEN1*; the catalytic component of the γ-secretase complex that produces the Aβ peptide). SAD is more common than FAD. While both types of AD can be associated with apolipoprotein E (ApoE) gene mutations, the SAD-specific etiology remains unknown [2]. A key element for determining the AD pathogenesis is to establish an effective disease model. Over the years, a large number of techniques, both in vivo and in vitro, have been used to model AD. However, none of these methods can fully mimic the pathological features observed in the brains of AD patients [3]. Usually, the in vivo methods involve animal models, mostly rodents, while the in vitro methods involve two-dimensional (2D) and 3D cultures of living cells (Fig. 1).

### AD animal models

There are three categories of AD animal (mostly murine) model: aging models, transgenic models (introduced with exogenous human-gene mutations), and models established by administration of AD-inducing agents.

Aging is an important AD pathogenic factor, and aging models can be divided into those of natural aging and those of rapid aging. Although the naturally aging mice may show some changes of the nervous system in their brain tissues, they have poor feeding ability and a high mortality. The rapid-aging model, SAMP8 mice from Kyoto University, show AD pathological characteristics [4], such as astrocytic response to oxidative stress and early-phase energy metabolic abnormalities, while other abnormalities such as NFTs and tau hyperphosphorylation are not present. In addition, subcutaneous or intraperitoneal injection of *D*-galactose can also cause metabolic disorders and learning and memory deficits in mice, but some factors such as the oxidative stress are different from those in naturally aging mice [5].

The discovery of AD genes has led to the rapid development of AD transgenic models. The onset and development of AD are related to genes such as *APP*, *PS* (encoding progenitor protein), and *ApoE*. Common transgenic mouse models include APP transgenic mice, PSEN1 transgenic mice, tau transgenic mice, APP/PSEN1 transgenic mice, and APP/PSEN1/tau transgenic mice. However, these models still suffer shortcomings. The APP transgenic mice do not have NFTs [6], tau hyperphosphorylation, or neuronal apoptosis [7], and the APP/PSEN1 transgenic mice show early Aβ deposition  and also gene expression instability, represented  by various appearance times and severities of Aβ deposition among the animals from different sources (e.g. from different companies) [8]. Gotz et al. [9] mutated the FT-DP-17 gene to create a tau-transgenic mouse model that demonstrates both AD behavior and NFTs but without Aβ deposition. The APP/PSEN1/tau transgenic mice have both Aβ accumulation and high levels of tau hyperphosphorylation, but they have poor survival [10]. Unlike human patients with AD in whom neuronal apoptosis occurs with increased age, in transgenic mouse models, brain atrophy is known to occur at early development and precedes the formation of Aβ accumulation [11–13].

In addition, approximately 90% of AD patients are SAD, which is thought to be stimulated by exogenous environmental factors. Murine SAD models have been established by injecting or feeding with harmful exogenous substances, including Aβ [14], aluminum [15], and streptozotocin [16]. However, none of these animal models show tau hyperphosphorylation.

Although the above-mentioned models recapitulate some pathological and behavioral features in AD patients, the levels of Aβ deposition and NFTs are still inconsistent. Overall, AD animal models have limitations including high mortality and poor representation of the structural, cellular and protein abnormalities in the human brain [17]. In addition, murine brain cortices are small and smooth, while human cortices are larger and have many sulci and gyri, which are closely related to the learning and memory abilities of humans [18]. Therefore, it is very difficult to mimic the human nervous system disease using animal models.

### AD cell models

Induced pluripotent stem cells (iPSCs) [19] (induced through fibroblast reprogramming) derived from FAD patients have been differentiated into neurons that show increased Aβ42 levels, supporting the idea that these FAD iPSCs can be a foundation for modelling FAD.

Compared to 2D cultures, 3D cell cultures have inherent advantages. First, compared to the 2D environment, cell-associated markers have higher expression and are more accurately localized in 3D cultures. In the 3D environment, synapses are shown to have complex morphologies (with 3D neurite extension) that better approximate what is seen within the body, with better localization of markers for nerve cells. Second, 3D cultures can more faithfully mimic the brain environment. For example, co-culture of neurons and glial cells could mimic the complex interaction between different cells in the brain, providing an important AD cellular model. But in 2D co-cultures of neurons and glial cells, cells can only spread horizontally rather than vertically. The upper layer of neurons grow above the base layer of glial cells, leading to an unexpected top-to-bottom polarity, which could be overcome in 3D co-cultures [20]. By visualizing an EGFP-fused wild-type tau protein in SH-SY5Y cell lines under both 2D and 3D conditions, Seidel et al. demonstrated a considerably higher level of this neuronal protein in the 3D cultures [21]. Additionally, a 3D environment allows cells to connect with each other. The 3D-plated neural cells are present as a bulbous mass with intercellular contacts in all directions, and are connected by neuronal synapses [21]. However, the 2D cultured cells are restricted to two-dimensional monolayers, and the neurite outgrowth and their interactions mainly occur in the horizontal plane of the substrate. This 2D spacing makes cell distance too large to form synaptic connections between cells and coordinated interactions. Thus, a suitable 3D microenvironment can sufficiently support neurite outgrowth to enable the formation of functional synapses between neurons, with neurites extending over several millimeters within the 3D bioactive scaffolding. This environment enables appropriate modelling of properties of specific neuronal subpopulations with distant target innervation. Neurons display complex 3D morphologies with rich neurite arborization in all dimensions, expression of mature cytoskeletal proteins, and demonstration of network connectivity [21]. Compared to the 3D conditions, no tight connections are observed between cells under 2D-culture conditions, and it is therefore not surprising that Aβ accumulations are difficult to occur in AD cells under 2D-culture conditions [22], because the Aβ produced after cell induction can spread to the culture medium and will be removed during medium replacement. The inability to produce Aβ aggregation makes it difficult to trigger subsequent pathogenic cascade reactions (e.g., NFTs and tau phosphorylation) [23].

Choi et al. [23] cultured for the first time human neural progenitor cells (hNPCs) over-expressing human *APP* or *APP* and *PSEN1* using a Matrigel substrate. In the 3D cultures, higher levels of toxic Aβ deposits and p-tau in hNPCs are detected compared to the 2D cultures. The combination of the two methodologies (i.e., iPSC-differentiated cells and 3D culture) demonstrates that an in vitro 3D environment can be a physiologically relevant and valid model for further studies of AD and for testing the efficacy of AD drugs [24]. The environment of a 3D hydrogel cell culture can provide a closed system for brain tissue and for nerve cells, which limits Aβ diffusion and provides an environment that favors Aβ deposition [25]. Ravi et al. [26] have argued that the 3D cell-culture conditions are advantageous over the 2D conditions due to their ability to provide spatial structuring, adhesion, proliferation, signaling, and mechanical cell transduction, which the 2D cell culture cannot provide [27]. Therefore, 3D cell culture is the key to exploring AD pathology [23]. These conditions also provide insights into the research and development of 3D tissues and organs.

### Development of 3D cerebral organoids

The brain is the most complex organ of the human body, containing complicated systems of the vasculature and neural connections. Studies of the human brain are limited in postmortem or biopsy tissues, and in vivo observations of pathological mechanisms in the central nervous system (CNS) have not been possible. The stem-cell technologies have been developed rapidly since the isolation of embryonic stem cells from murine embryos in 1981 [28]. The stem cells can develop into intact organoids because of their ability to self-organize [29–32].

Numerous in vitro tissue-culture studies, in combination with the use of human iPSCs (hiPSCs), have provided a solid foundation for 3D neural tissue models [33]. In 1998, Thomson et al. [34] isolated human embryonic stem cells and then differentiated them into various types of neural cells, including neural stem cells and embryonic ganglion cells. In 2007, Takahashi et al. [35] successfully induced hiPSCs from differentiated and mature human somatic cells by reprogramming the expression of four transcription factors (Oct3/4, Sox2, Klf4, and c-myc) in the latter cells. With further development of stem cell technologies, these differentiated and mature human somatic cells can now be differentiated into embryoid bodies, neural rosettes, and 3D neuroepithelial tissues. The neuroepithelial tissues may have some genetic elements of the human brain, but they lack a brain structure and coordination between different subregions of the brain [36]. However, in 2011 a 3D neuroepithelial tissue that displays intact tissue architecture was created for the first time by Eiraku et al. [32]. This neural tissue, maintained in a floating environment, has the ability to both self-organize and self-develop to ensure subregional architectures. Sato et al. [29] then developed a gel-like extracellular matrix called Matrigel, which has been shown to provide an appropriate environment for self-organization of a variety of organoids, including the stomach, liver, lung, and kidney organoids [37–40]. In 2013, Lancaster et al. [41] differentiated hiPSCs for the first time into a whole cerebral organoid based on the methods of the studies described above. These hiPSCs differentiated into a neuroectoderm structure, simulating an early development stage of the human embryonic cerebral cortex [42]. They further modelled human microcephaly in cerebral organoids using the patient-specific iPSCs, and reported that the microcephaly organoid is significantly different from cerebral organoids derived from normal human stem cells. The patient organoids show very few progenitor regions, decreased radial glial stem cells and increased neurons, suggesting premature neuronal differentiation, a defect that can explain the disease phenotype [41]. The results indicate that the human cerebral organoids can have the same genetic characteristics as the stem-cell providers, and can recapitulate both development and disease. Using RNA sequencing technology, Dang et al. [43] found that 30-day-old human cerebral organoids have the same pattern of gene expression as that in fetal brains at 8–9 weeks of pregnancy.

**Fig. 2:**
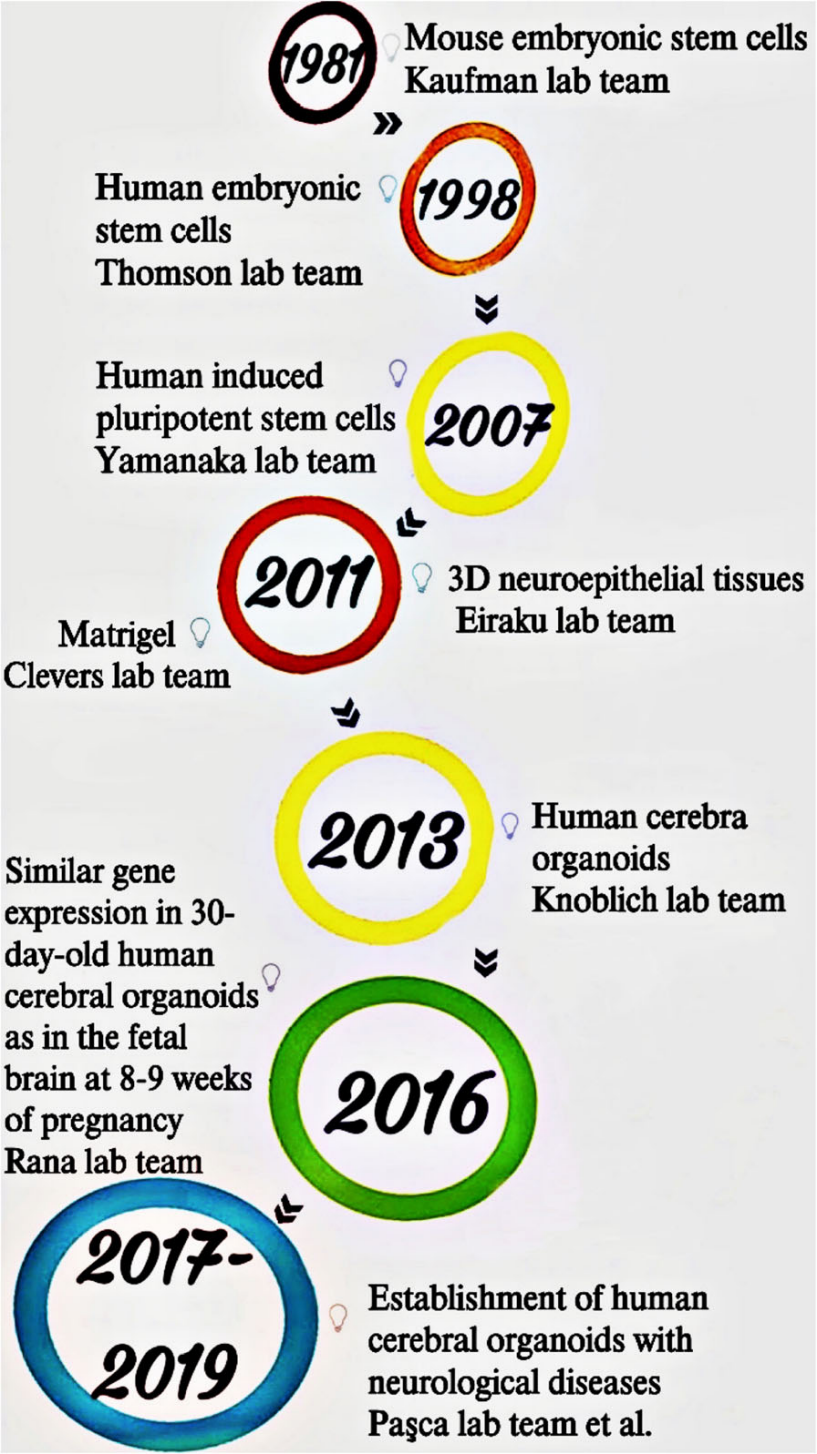
The development of 3D cerebral organoids. Stem-cell technology was developed in 1981; human embryonic stem cells were successfully differentiated into various types of neural cells (e.g., neural stem cells and embryonic ganglion cells) in 1998; mature human somatic cells were reprogrammed into human stem cells in 2007; Matrigel, a gel-like extracellular matrix, became available in 2011. These developments were prerequisites for the emergence of cerebral organoids. In 2013, Lancaster et al. first used hiPSCs for whole-cerebral organoid differentiation. Later, Dang et al. reported that 30-day-old human cerebral organoids have similar gene expression as in the fetal brain (8–9 weeks of pregnancy). Subsequently, a number of CNS disease models were developed based on cerebral organoids

Currently, protocols for the generation of organoids with characteristics of various human brain regions, including the cortex [44], basal ganglia [45], hippocampus [46], choroid plexus [47], thalamus [48], retina [31], striatum [49], hypothalamus [50, 51], midbrain [51–53], cerebellum [54, 55], and human spinal cord [56, 57], have been reported. The use of bioreactor has also improved the differentiation of organoid cultures. For example, Qian et al. [51] designed a miniaturized spinning bioreactor that is cost-effective and simple to use to improve oxygen diffusion and nutrient distribution for the generation of forebrain, midbrain, and hypothalamic organoids from human iPSCs. This bioreactor is a promising development for the use of organoids for both modeling brain development and disease and for therapeutic screening. The history of development of cerebral organoids is shown in Fig. 2.

The cerebral organoids have great clinical value as they can recapitulate the developmental processes and structural characteristics in early embryonic brains and have human-specific genotypes and protein expression patterns [58]. First, the area expansions and folded sulcus of the cerebral organoid cortex are similar to the human cerebral cortex. During the development of human cerebral organoids, both the ventricular zone and the later-formed sub-ventricular zone expand markedly and increase in the overall size, and this enlargered surface area is organized into continuous cortical folds. This in vitro expansion is triggered by increased cell proliferation, transiently delayed neuronal differentiation, and the overall amplification of the neural-progenitor population. This type of amplification is similar to the cellular and anatomical framework of the developing human cortex [59]. Second, cerebral organoids demonstrate an abundance of nerve collaterals and synaptic connections. Third, the genetic background and cellular/structural compositions of cerebral organoids are similar to those of the human brain [60, 61]. Overall, the cerebral organoids have shown great potential for creating accurate models of CNS diseases, including AD, and provide a platform for validating disease mechanisms. Subsequent cerebral organoid studies have focused on a variety of CNS diseases, such as brain developmental diseases, CNS infection, epilepsy, and CNS tumors [45, 51, 59, 62, 63].

### AD cerebral organoids

There are three methods commonly used to model AD in cerebral organoids: using Aftin-5 (an Aβ42 agonist) to induce Aβ42 peptide production in cerebral organoids, which resembles the SAD human brain [64]; producing cerebral organoids from iPSCs derived from FAD patients [65, 66]; and converting APOE3 to APOE4 in SAD patient-derived iPSCs to create differentiated SAD cerebral organoids [67].

Qualified AD cerebral organoids should manifest with characteristics of AD, including Aβ peptide, hyperphosphorylated tau protein, NFTs, synaptic dysfunction, neuronal degeneration and neuroinflammation [68].

### FAD cerebral organoids

Several recent studies have shown potentials of patient-derived iPSCs to model diverse aspects of AD, such as higher levels of more amyloidogenic variants of the Aβ protein, increased tau phosphorylation, higher susceptibility to Aβ toxicity, and the presence of cellular stress markers [69–78].

Raja et al. [66] have obtained pluripotent stem cells from FAD patients harboring *APP* duplications or *PSEN1* mutations and differentiated them into FAD cerebral organoids. These organoids demonstrate both amyloid and tau pathologies, and more powerfully, this model shows a distinct time line: the increases in P-tau levels in these organoids occur later than the Aβ aggregates, similar to what occurs in the human FAD pathological process. In contrast, most mouse models of AD must carry multiple transgenes in order to achieve robust amyloid phenotypes and they rarely have significant tau pathology or neuronal loss. Gonzalez et al. [65] also created a different type of FAD cerebral organoid using patient stem cells carrying a missense mutation (A246E) in the *PSEN1* gene linked to early-onset AD. These FAD cerebral organoids exhibit the same Aβ and P-tau protein aggregates as reported by Raja et al. [66], and show a degree of cellular apoptosis and NFTs that is proportional to the accumulation of protein aggregates. The two types of FAD cerebral organoids were cultured for 90 days [66] and 110 days [65], respectively. In addition, the study of Raja et al. [66] did not mention cellular apoptosis or NFTs in the organoids, therefore it is unclear if these alterations occur in this type of cerebral organoid.

Yan et al. [79] also used stem cells with *PSEN1* mutations from FAD patients to produce FAD cerebral organoids. These organoids show Aβ aggregation, increased P-tau, and cellular apoptosis. In addition, the cerebral organoids have the following additional features: (1) glutamatergic neurons are decreased in 17-day-FAD cerebral organoids but increased in 28-day-FAD cerebral organoids, which is consistent with a vicious cycle of Aβ-dependent neuronal hyperactivation in mouse models of Aβ-amyloidosis reported by Zott et al. [80]; (2) lactate dehydrogenase activity is high in FAD cerebral organoids, which causes toxicity to nerve cells directly; (3) gene expression of inflammatory factors interleukin 6 and tumor necrosis factor alpha is greatly increased in FAD cerebral organoids, which can induce astrocytic toxicity and neuronal apoptosis; and (4) matrix metalloproteinase-2 (MMP2) and MMP3 are downregulated and syndecan-3 is upregulated in the FAD cerebral organoids. The extracellular matrix (ECM) is fundamental for nerve cells in the human CNS, providing a variety of biochemical signals for their generation, migration, differentiation, and synaptic plasticity. Of the main ECM enzymes, MMP2 is known to play an important role in neurogenesis, basement membrane remodeling, and axon regeneration; MMP3 can promote synaptic remodeling and degradation of Aβ protein. Syndecan-3 is a member of the heparan sulfate glycoprotein family known to mediate cell proliferation, axon growth, and inflammatory processes. The alterations of the three molecules demonstrate that neuroinflammation, apoptosis, and synaptic degeneration may be related to the changes in ECM components and remodeling during the development of AD.

Arber et al. [81] observed accelerated aging and a predisposition for neurodegeneration in FAD cerebral organoids derived from iPSCs harboring *PSEN1* mutations. In mutation-confirmed postmortem tissues, AD cases show mutation-specific effects and a trend toward a reduction in newborn neurons, supporting a premature-aging phenotype. Overall, these results support the idea of altered neurogenesis resulting from FAD mutations, and suggest that the neural stem-cell biology is affected by both aging and disease.

### SAD cerebral organoids

Pavoni et al. [64] used Aftin-5 (an Aβ42 inducer that increases the production and secretion of soluble extracellular amyloid peptides) to stimulate cerebral organoids derived from normal human stem cells to create SAD cerebral organoids. The Aftin-5 treatment led to a reproducible disruption of the physiological balance between Aβ42 and Aβ40 observed during normal maturation. The concentration of secreted Aβ42 and the Aβ42:Aβ40 ratio increased during the 4 days of treatment, whereas the concentration of secreted Aβ40 remained stable. Within this timeframe, neither the phosphorylation of tau protein nor the extracellular localization of Aβ was shown using immunohistochemistry in the Aftin-5-treated organoids, and Aβ aggregation in the SAD cerebral organoids appeared to develop in a time-dependent manner similar to the FAD cerebral organoids.

Lin et al. [67] have used CRISPR/Cas9 to induce APOE3-to-APOE4 mutation into human pluripotent stem cells. This mutation has been identified as the greatest single genetic risk factor for SAD. The level of APOE protein in the APOE4 organoids is significantly lower than that in normal organoids, and the two organoid types have similar total numbers of cells. Increased levels of both Aβ and P-tau in the APOE4 organoids compared to the APOE3 organoids become apparent after 6 months of culture, which is relatively delayed compared to that seen after 2 months of culture in organoids with FAD mutations [66]. Increased numbers of synapses, early endosomes, and compromised Aβ uptake by astrocytes also occur in the APOE4 cerebral organoids. Neuronal Aβ secretion may have contributed to the Aβ accumulations observed in APOE4 organoids cultured for 6 months. Therefore, the APOE4 variant alone is sufficient to cause AD hallmarks in cerebral organoids.

A powerful aspect of this model is the spontaneous appearance of both amyloid and tau pathology, and the distinct timeline of their appearance: the Aβ deposits precede the tau pathology. In contrast, as mentioned above, the inclusion of both of these facets of AD in mouse models is challenging; most models carrying multiple transgenes to achieve robust amyloid phenotypes rarely demonstrate significant tau pathology or neuronal loss.

Zhao et al. [82] have produced SAD cerebral organoids using human pluripotent stem cells derived from AD patients carrying *APOE* gene mutations. These SAD cerebral organoids exhibit high levels of both Aβ and P-tau, neuronal apoptosis, synaptic loss, and increased formation of stress granules, indicating that APOE4 is independently associated with increased levels of P-tau in iPSC-derived cerebral organoids.

### Problems with current AD cerebral organoids and possible solutions

#### Lack of microglia

The current AD cerebral organoids contain neurons and neuronal progenitor cells (derived from the ectoderm) but lack microglial cells (derived from the mesoderm) which play an important immune-system role during AD development [42]. The activation of microglial cells induced by Aβ occurs early in the AD pathological process. Microglial cells stimulated by Aβ can release a variety of inflammatory mediators, causing neuronal injury/death, synaptic damage, and neurotransmitter changes. Proliferation and activation of microglial cells occurs throughout the AD pathological process [83].

At the time of this review, some researchers have already made cerebral organoids containing microglial cells. Ormel et al. [84] have created cerebral organoids containing microglia by improving the cerebral organoid culture formulation reported by Lancaster [42]. Nzou et al. [85] have attempted to combine human microglial and microvascular endothelial cells, peripheral nerve cells, astrocytes, and oligodendrocytes to form an organoid with a brain barrier that includes microglia. Song et al. [86] have differentiated hiPSCs into isogenic microglial cells and cerebral organoids and then combined these cells to enhance the inflammatory response of the cerebral organoids. These studies suggest that the AD cerebral organoids containing microglia can be made by integrating and co-culturing different cells, and changing the culture formulations.

#### Lack of vascular system

The non-vascular AD cerebral organoids have many disadvantages. First, although the genetic coding in cerebral organoids is very similar to that observed in the fetal brain [87], which is considered as a valuable proof for their application to mimic the human fetal brain, the use of this model to simulate age-related diseases is limited, because cerebral organoids may demonstrate volume shrinkage and internal cellular apoptosis after 180 days of culture, as the nutrients and oxygen cannot enter the innermost organoid regions without a vascular system. Thus, AD cerebral organoids cannot simulate the real AD brain well because they are not “old” enough [33]. Second, the brain vasculature plays an important role in the accumulation and clearance of neurotoxins during AD pathogenesis [88]. The neurovascular unit, which includes the vasculature (vascular endothelial cells, perivascular cells, and vascular smooth muscle cells), glial cells (astrocytes, microglia, and oligodendroglia) and neurons, is fundamental to the blood-brain barrier. This barrier not only supplies essential energy and nutrients to sustain neurons and their synaptic functions, but also limits the entry of some blood components, including red blood cells, white blood cells, and neurotoxicants into the brain. These components of the neurovascular unit work together to sustain the normal permeability of the blood-brain barrier. When the blood-brain barrier is blocked, various neural and vascular toxic substances may accumulate in the brain, resulting in pathological changes, such as cerebral edema, microcirculation disorder, microthrombus, and cerebral hypoperfusion. Third, the brain hemodynamics is closely related to Aβ production and tau phosphorylation. Obstacles for brain microcirculation and disorders of hemodynamics can lead to APP synthesis and its increased expression, resulting in Aβ deposits [89]. Moreover, the phosphorylated tau protein is known to increase in the presence of low cerebral blood perfusion [89]. Fourth, the lack of a blood-brain barrier in organoids makes the development of anti-AD drugs challenging, as the permeability to anti-AD drugs in cerebral organoids cannot truly simulate their diffusion and absorption in the real AD brain.

Several attempts have been made to obtain cerebral organoids with a vascular system. Mansour et al. [90] have implanted a mature human cerebral organoid into the mouse brain in vivo, and the organoid grafts demonstrated integration, good viability, long-term survival, vascularization, functional neuronal activity, and synaptic connectivity with the host. Pham et al. [91] have used differentiated human pluripotent stem cells to form vascular endothelial cells and cerebral organoids. They further combined these cells with Matrigel, vascular endothelial growth factor, and other growth factors in the culture medium on day 34 of culture; a vascular system then appeared 3–5 weeks afterwards. Cakir et al. [92] used CRISPR/Cas9 gene editing to make human embryonic stem cells express the human vascular endothelial gene ETS variant 2 (*ETV2*), then they combined the normal human embryonic stem cells and ETV2-embryonic stem cells in a 1:5 ratio to create human cerebral organoids with a vascular system.

#### Lack of connections with other organs

The brain is not independent from the rest of the body. It has close relationships with a variety of organs, and similarly, the AD brain pathogenesis also has close relationships with other organs. This can be illustrated by connections between the brain and the gut, kidneys, and heart as examples.

Gut microbiota communicate with the brain *via* the gut-brain-axis. The bidirectionality of this axis is often demonstrated by regulations of the gastrointestinal system and the CNS. Imbalances of intestinal flora can stimulate host immune responses and promote a variety of inflammatory diseases [93]. Recently, a considerable amount of evidence has supported that intestinal dysbacteriosis is closely associated with the development of AD [94], and Minter et al. [95] have found that disruptions of intestinal microbial diversity could induce both neuroinflammation and amyloidosis. Calvani et al. [96] have also demonstrated that the lipopolysaccharides produced by intestinal microbes are present in the brains of patients who died of AD. Recent novel AD drugs, such as sodium oligoamines (a low- molecular-weight acid oligosaccharide compound extracted from marine brown algae), may alleviate neuroinflammation and reverse cognitive decline in AD patients by suppressing gut dysbiosis and associated phenylalanine/isoleucine accumulations [97].

Similar to the gut-brain-axis, the brain and the kidney communicate with each other in the brain-intestine-kidney axis [98]. Immune cells developing in the bone marrow are activated by the gut microbiome and enter the bloodstream, thereby causing an inflammatory response to the kidneys. Alterations of the intestinal flora not only affect the brain directly but also activate inflammatory response from the sympathetic nervous system throughout the body, causing hypertension and renal damage [99].

A meta-analysis has shown that the AD morbidity in patients with chronic kidney disease is up to 39% compared to that in normal people [100], and proteinuria levels have been shown to be closely correlated with the cognitive impairment [101]. The development of cognitive impairment may be due to the following reasons. First, urea nitrogen and creatinine both can cause impairment to nerve cells directly. Short-term increases in urea nitrogen and creatinine may cause uremia encephalopathy, language confusion, mental abnormalities, and even coma in patients. Long-term increase of both urea nitrogen and creatinine can cause continuous toxicity to nerve cells, microglial activation and neuronal apoptosis [102]. Clinical studies have shown that the concentrations of urea nitrogen and creatinine in the cognitive brain areas of chronic kidney disease patients are up to 10 times higher than normal concentrations [103]. Second, electrolyte imbalances can cause cerebral edema and insufficient oxygen supply to the brain, which is a direct cause of cognitive impairment [104]. Third, hypertension is a common complication of chronic kidney disease and can make cerebral arterioles both twisted and thin. Under the influence of hypertension, cerebral infarctions or hemorrhages may occur, which damage the brain parenchyma and cause cognitive impairment. Therefore, renal insufficiency is an independent risk factor for both cognitive impairment and dementia [101].

Based on previous reports, heart failure is significantly related to patient cognitive impairment [105–107]. Cerebral hypoperfusion can be caused directly by cardiac insufficiency. One important determinant of cerebral perfusion is cerebral vascular radius, which is regulated by many neurohormones and body fluids. The mediators of vasodilation include bradykinins, nitric oxide, potassium, and magnesium, while the mediators of vasoconstriction include norepinephrine, serotonin, calcium, arterial hypoventilation, and hyperoxygenation. Under normal conditions the automatic vasoregulation can balance these mediators to meet the brain’s perfusion needs; however, dysregulation of the vasodilation/vasoconstriction mediators caused by cardiac insufficiency can make microvascular structures narrow and twisted, thereby obstructing the central circulatory system. The frontal lobes, areas closely associated with cognitive functions, are particularly vulnerable to hypoperfusion and often suffer neuronal damage in patients with heart insufficiency.

It is therefore necessary that qualified AD cerebral organoids satisfy the integrity of tissues and cells as well as the connections with other organs and connections in the whole body. In vivo organoid transplantation experiments [90] have demonstrated that the embedded cerebral organoid can form connections and circuits with its murine host, grow blood vessels in the implant, and have neuronal synapses and glial cells fused with the mouse brain. This fusion functionally results in a connection between the transplant, the host brain, and other organs. Notably, the implanted human cerebral organoid demonstrates the same developmental processes and characteristics of neurogenic dynamics as the host grows.

**Fig. 3:**
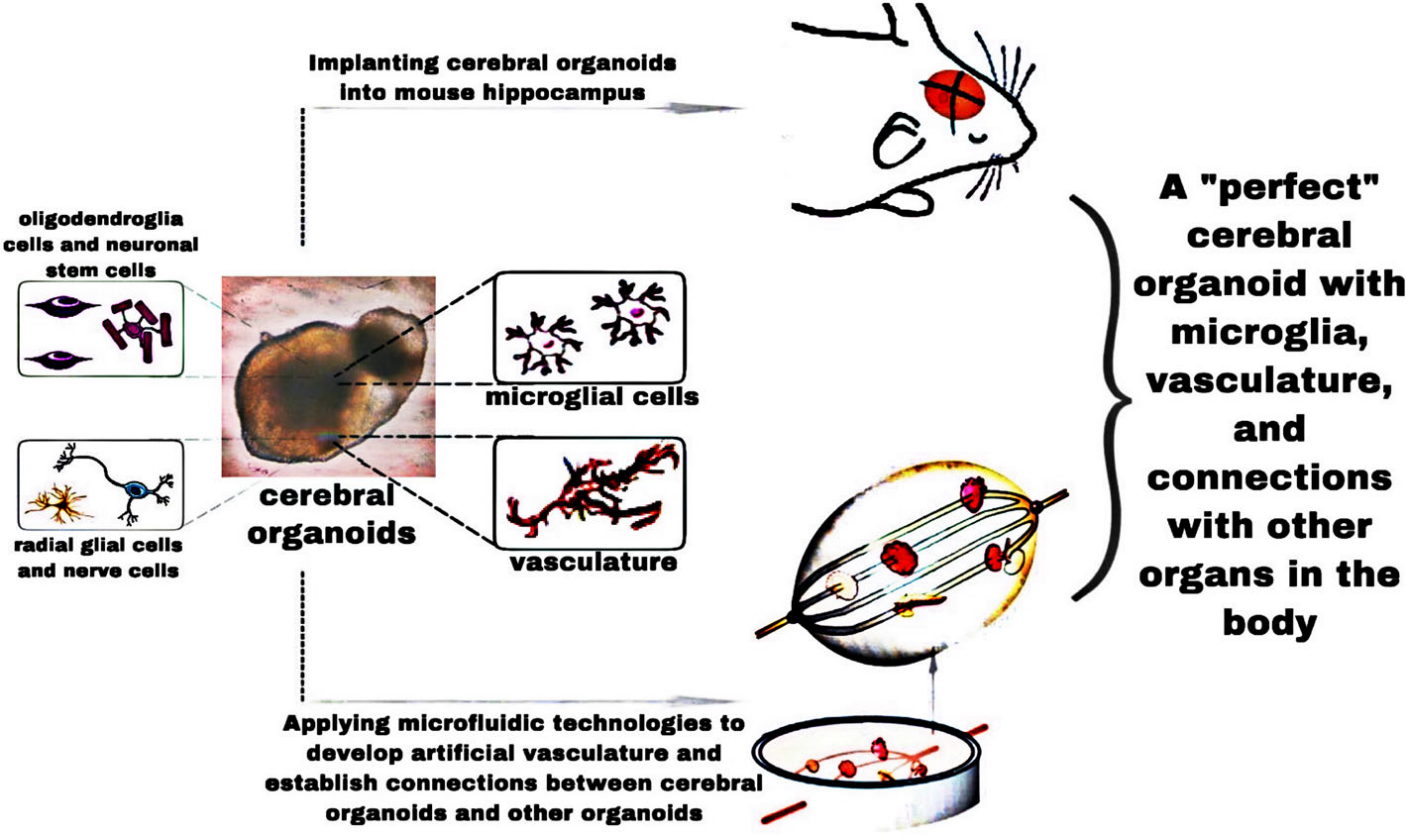
Establishment of a qualified AD cerebral organoid. Implantation of exogenous microglia, use of the cerebral organoid fusion technology, and addition of microfluidics are suggested protocol improvements for establishing qualified AD cerebral organoids

Recently, microfluidic technologies have been used to develop an artificial vascular system [108]. Incorporating cerebral organoids and microfluidic chips may be a way to ensure that signaling molecules, nutrients, and oxygen can reach their innermost regions. The use of microfluidic chips can extend the survival of many types of organoid to obtain “older” organoids [109]. Moreover, microfluidic technologies can be used to establish connections between cerebral organoids and other kinds of organoids in the future (Fig. 3).

### Application of novel cerebral organoids in AD research

**Fig. 4:**
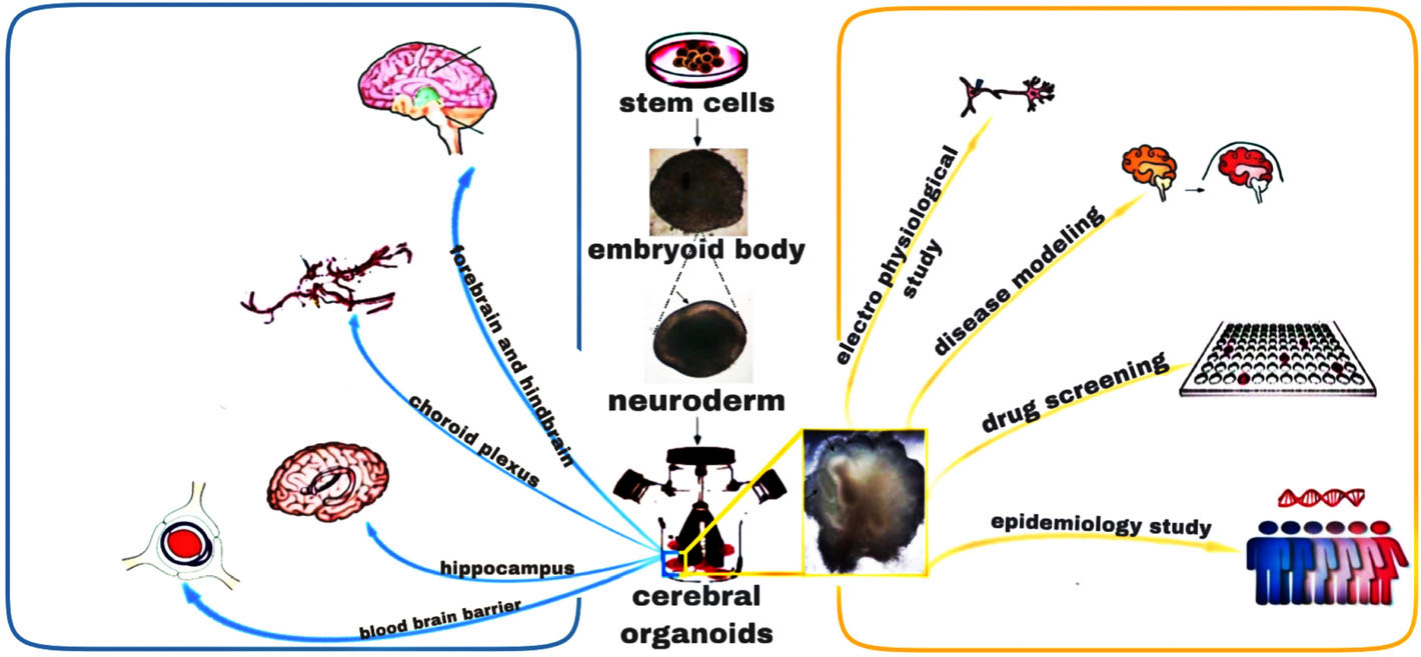
Applications of AD cerebral organoids. Qualified AD cerebral organoids may contain a variety of neuronal cells, a blood-brain barrier, and connections with other areas. These features can be used to test the blood-brain barrier permeabilities of drugs and compare them to the human brain permeabilities, to explore cerebral electrophysiology and connections between the brain and other organs, to study human diseases in the central nervous system (not just AD), and for epidemiological investigations

Better-qualified AD cerebral organoids will not only accelerate the exploration of anti-AD drugs but also provide an unprecedented boost for exploring the mechanisms underlying AD pathogenesis (Fig. 4).

First, cerebral organoids can provide a better approach for understanding the etiology and pathogenesis of AD. In the coronavirus disease 2019 pandemic caused by the severe acute respiratory syndrome coronavirus 2 (SARS-CoV-2), by using cerebral organoids, Ramani et al. [110] have reported that the preferential target of SARS-CoV-2 is the cortical plate rather than the neural stem cells. The virus also induces neuronal death and tau phosphorylation, and triggers a cascade of downstream effects that could initiate AD-like diseases.

Second, the qualified AD cerebral organoids may provide an unprecedent boost for the exploration of new anti-AD drugs. Yan et al. [79] have evaluated potential anti-AD drugs (γ-secretase inhibitor DAPT, heparin, and heparinase III) in FAD cerebral organoids, and found that DAPT inhibits endogenous Aβ42 aggregation, reduces cytotoxicity, and improves neuronal survival; heparinase III selectively degrades heparan sulfate proteoglycans (HSPGs) in the ECM and reduces the binding between proinflammatory factors and HSPGs, preventing the Aβ-HSPG interactions. The heparinase III also decreases the intracellular tau aggregation.

In addition to mimicking the human genetic background and cellular/tissue morphologies, qualified AD cerebral organoids can have blood-brain barriers and connections with other organs. These features can be used to explore new AD treatments, as the presence of blood-brain barrier enables drug developers to measure the permeabilities of different anti-AD drugs. Furthermore, the connections between the brain and other organs can be modeled using organoids, thus the hormones, cytokines secreted by other organs, or specific intestinal flora may be a future therapeutic direction for AD. Given that the cerebral organoids are cultures with isolated-organ survival, these human-like brain models may allow for direct-observation experiments on anti-AD drugs (e.g., distribution of drugs with distinguishable colors).

Third, cerebral organoids by themselves may be a treatment for AD. Kitahara et al. [111] have grafted 10-week-old human cerebral organoids in the cynomolgus monkey brain, and found that the cerebral organoids can survive in the host cerebral cortex and provide axonal extensions along the callosal and sub-cerebral projections. This study suggests that this use of cerebral organoids may be a novel approach to reconstructing neural circuits after AD neuronal death in the cerebral cortex. Similarly, in the study of Dong et al. [112], the cerebral organoids transplanted into the murine brain acquire both electrophysiological maturity and synaptic connections with host neurons. In addition, the host animals have stronger auditory startle/fear responses, suggesting that the grafted cerebral organoids can contribute to neuronal regeneration.

Cerebral organoids may also play a role in epidemiological studies. Stem cells can be obtained from patients from different geographic regions and with different races and then be differentiated into cerebral organoids. This is a feasible method for obtaining more AD information (e.g., specific characteristics of cerebral pathologies) in different populations.

### Ethical issues on cerebral organoids

For more than a decade, efforts have been made to build tissues, organ-like structures, and even whole animals from cells. Although the brain, retina, intestine, kidney, pancreas, liver, inner ear, and thyroid can all be made, ethical issues are only concerning the brain. The human cerebral organoids derived from stem cells have the ability to self-assemble just like in the developing brain, and using the single-cell RNA sequencing technology, Camp et al. [87] have found that the genetic coding in cerebral organoids is very similar to that observed in the fetal brain.

At present, there are two main ethical issues on cerebral organoids: the first involves the source of stem cells and the second involves whether cerebral organoids are conscious and can feel pain. The stem-cell sources are entirely manageable, as investigators must obtain consent from stem-cell providers and complete ethical reviews. However, it remains debatable whether cerebral organoids are conscious entities that can feel pain. Tsutomu et al. [113] have argued that cerebral organoids without means for sensation cannot be self-conscious, phenomenal conscious, or even access consciousness. Without sensory input and motor output, phenomenal consciousness cannot occur, and access to consciousness and self-consciousness are not possible. Accordingly, current cerebral organoids are unlikely to be conscious entities.

## Conclusion

Cerebral organoids have contributed to our understanding of the pathogenesis of nervous system diseases that cannot be cured, such as AD, and will continue to play important roles in the development of modern medicine. Improvement in methods for developing cerebral organoids will provide new directions for studies on CNS diseases, including AD.

## Data Availability

Not applicable.

## Abbreviations

AD: Alzheimer’s disease
Aβ: Amyloid-β
NFTs: Neurofibrillary tangles
FAD: Familial Alzheimer’s disease
SAD: Sporadic Alzheimer’s disease
APP: Amyloid precursor protein
PSEN1: Presenilin 1
ApoE: Apolipoprotein E
hiPSC: Human induced pluripotent stem cell
APOE3: Apoliproprotein-3
APOE4: Apoliproprotein-4
CNS: Central nervous system
ETV2: ETS variant 2
ECM: Extracellular matrix
MMP2: Matrix metalloproteinase-2
MMP3: Matrix metalloproteinase-3
DAPT: γ-secretase inhibitor
HSPG: Heparan sulfate proteoglycan
SARS-CoV-2: Severe acute respiratory syndrome coronavirus 2
